# Supplementary material for: Data-driven understanding on soccer team tactics and ranking trends: Elo rating-based trends on European soccer leagues
Source: PLoS One. 2025 Feb 5;20(2):e0318485. doi: 10.1371/journal.pone.0318485 (PMC11798493; doi:10.1371/journal.pone.0318485)
Supplement: Attachment — Submitted filename: Review of Research Paper.pdf [file pone.0318485.s002.pdf]

PLOS ONE : A Peer -Reviewed, Open Access Journal: Reviewers Guide

SECTION I	Details
**Reviewer's Name**	Dr. D.Kalidoss
**E-mail**	dr.kalidoss@kalingauniversity.ac.in
**Manuscript Number**	PONE-D-24-17238R1
**Title**	Data-driven understanding on soccer team tactics and ranking trends: Elo rating-based trends on European soccer leagues
**Date Sent to Reviewer**	17th November 2024
**Date Expected from Reviewer**	
**Specialization**	Physical Education and Sports

SECTION II: Comments per Section of Manuscript

Section	Comments
**General Comment**	The paper addresses an important and emerging field in sports analytics, particularly soccer. By focusing on team trends and strategy prediction, it contributes to advancing the use of data science in sports. The study is well-structured and provides clear research questions. However, there are areas where clarity and depth could be improved, particularly in explaining the methodological approaches and integrating findings with practical applications.
**Introduction**	The introduction provides a clear overview of the significance of soccer analytics and highlights the role of big data and machine learning in advancing sports analytics. However, A brief explanation of the Elo rating system and its relevance to team performance could strengthen the context.
**Methodology**	The qualitative methodology is suitable for the study, the dual approach of count-based and rank-based methods provides a comprehensive framework for analyzing strategies but more clarity on dataset specifics (e.g., sample size, range of seasons analyzed, preprocessing steps) would enhance replicability.
**Results**	Results provides quantitative findings, such as the accuracy of predictions (85% for defensive strategies and 89% for aggressive strategies), but Lack of comparative benchmarks with other models or methods makes it difficult to evaluate the study's innovation.
**Discussion**	The discussion highlights the practical implications of predictive models for coaching and game preparation, but it could delve deeper into the limitations of the study, such as potential overfitting or generalizability issues.

Section	Comments
**Bibliography/References**	References are diverse and relevant, covering foundational concepts and recent advancements in sports analytics, but some references appear dated; more recent studies could be incorporated to reflect current trends in machine learning and sports analytics.
**Others**	The study's focus on trend-based strategy prediction is novel and valuable, but the lack of practical implementation diminishes its immediate impact.
**Decision**	The study is a valuable contribution to soccer analytics, but certain areas need refinement, particularly in methodology transparency, results presentation, and discussion depth.

SECTION III - Please rate the following

Criteria	Rating (1 = Excellent, 4 = Poor)
**Originality**	2 (Good)
**Contribution to the Field**	2 (Good)
**Technical Quality**	2 (Good)
**Clarity of Presentation**	3 (Fair)
**Depth of Research**	2 (Good)

SECTION IV - Recommendation

Recommendation	Mark with an X
Accept as it is	
Requires Minor Corrections	
**Requires Moderate Revision**	**X**
Requires Major Revision	
Submit to Another Publication such as	
Reject on Grounds of (Please be Specific)	

SECTION V: Additional Comments

The manuscript demonstrates a robust methodology and significant insights but could benefit from a clearer articulation of its broader implications. For instance, explicitly state how this research can influence team preparation or scouting processes.

- **Data Usage**: The dataset's origin (matches from the top five European soccer leagues) is stated, but additional details about the dataset are required.

- **Methodology Detail**: Explain how defense and attack scores were computed mathematically for the count-based method. Additionally, Include metrics or visualizations that validate the quality of the clusters (e.g., silhouette scores).
- **Results Section**: The results are promising (85% and 89% prediction accuracies), but Include a detailed breakdown of these results, such as confusion matrices or metrics like precision, recall, and F1-score for different strategies.
- **English Corrections**: The manuscript contains grammatical errors that need to be corrected. Refine transitions between sections to improve readability.
- **Conclusion**: The conclusion could be clearly summarize the key findings (e.g., superiority of rank-based predictions for offensive strategies) and emphasize the practical implications of this research for coaches, analysts, and other stakeholders in professional soccer.
