# Supplementary material for: Data-driven understanding on soccer team tactics and ranking trends: Elo rating-based trends on European soccer leagues
Source: PLoS One. 2025 Feb 5;20(2):e0318485. doi: 10.1371/journal.pone.0318485 (PMC11798493; doi:10.1371/journal.pone.0318485)
Supplement: Attachment — Submitted filename: Revision-Letter.docx [file pone.0318485.s001.docx]

**Journal Revision Letter**

Dear **reviewers**

Thank you very much for your comments and suggestions on our paper titled **“Data-driven understanding on soccer team tactics and ranking trends: Elo rating-based trends on European soccer leagues”**. The comments are very insightful and helpful in improving the quality of our paper and provide important guidance to our research. We have carefully considered and paid special attention to every comment and have responded to each one of them as detailed below. We sincerely hope that the revisions will be satisfactory for your approval.

Here is a point-by-point response to the reviewers’ comments and concerns.

**Reviewer 1**

| Comments | Responses |
| --- | --- |
| 1. *Provides good overview, though with some grammatical errors, and lacks details about the methodology. “Using a dataset comprising matches from the top five European soccer leagues, we analyze team performance trends over time using the Elo rating system and rolling regression.” should be “Using a dataset comprising matches from the top five European soccer leagues, we analyzed team performance trends over time using the Elo rating system and rolling regression.”.* | Thank you for your comment. I have revised the abstract to address the grammatical errors you pointed out. Additionally, I have included more detailed information about the methodologies to address the lack of specificity. Here are the improved sections:  Original Sentence: “Using a dataset comprising matches from the top five European soccer leagues, we analyze team performance trends over time using the Elo rating system and rolling regression.”  Revised Sentence: “Using a dataset comprising matches from the top five European soccer leagues, we analyzed team performance trends over time using the Elo rating system and rolling regression.”  Revised Sentence: “For the count-based method, the number of forwards, midfielders, and defenders is used to calculate respective defense and attack scores. For the rank-based method, teams are classified into various levels based on their rankings, and strategies are evaluated accordingly.” |
| 2. *There are some areas with too few references, and the current references could be more varied and recent..* | Thank you for your comment. I agree that some areas of the paper had too few references. As you suggested, it is important to include a wider variety of references. We have added additional references to the paper. Below are the revisions made:  The added references are as follows:   1. Lasek J, Szlávik Z, Bhulai S. The predictive power of ranking systems in association football. 2013;1(1):27–46. 2. Rein R, Memmert D. Big data and tactical analysis in elite soccer: future challenges and opportunities for sports science. 2016;5:1–13.   The detailed revision is written from line 132 and line 247. |
| 3. *Introduction: Well-structured, though with the potential for further detail and more recent references.“The main goal of this research is not to directly assist coaches’ decision-making by predicting specific teams’ tactics and game outcomes, but rather to forecast strategies based on team trends, thereby aiding their game preparation process.” should be revised for clarity: “The main goal of this research is to forecast team strategies based on trend analysis, thereby aiding coaches in their game preparation process, rather than directly predicting specific teams’ tactics and game outcomes.”* | Thank you for your comment. I agree with your perspective and realize that I may not have made this point clear enough. As you noted, the primary aim of this research is not to directly predict specific teams' tactics and game outcomes, but to assist in game preparation by forecasting strategies based on trend analysis. For clarity, I have revised the content accordingly.  Original Sentence: “The main goal of this research is not to directly assist coaches' decision-making by predicting specific teams' tactics and game outcomes, but rather to forecast strategies based on team trends, thereby aiding their game preparation process.”  Revised Sentence: “The main goal of this research is to forecast team strategies based on trend analysis, thereby aiding coaches in their game preparation process, rather than directly predicting specific teams’ tactics and game outcomes.”  The detailed revision is written from line 14 to line 17. |
| 4. *Add recent references for claims about the impact of big data and AI on sports analytics. Moreover, plos one does not consider reviews. The manuscript presents an interesting study but requires major revisions to enhance clarity, detail, and academic rigor.* | Thank you for your comment. I agree that there was a lack of supporting materials regarding the impact of big data and AI on sports analytics. To address this, we have added references to support our statements.  “The advent of big data and artificial intelligence (AI) has profoundly transformed sports analytics. Recent advancements in big data analytics have paved the way for more detailed sports analyses, particularly in soccer.” We have added recent references to this sentence.   1. Thakkar P, Shah M. An Assessment of Football Through the Lens of Data Science. Springer Science and Business Media LLC; 2021. p. 823. (Annals of Data Science; vol. 8). 2. Román-Gallego J-Á, Pérez-Delgado M-L, Cofiño-Gavito F-J, Conde MÁ, Rodríguez-Rodrigo R. Analysis and Parameterization of Sports Performance: A Case Study of Soccer. MDPI AG; 2023. (Applied Sciences; vol. 13). 3. Muhammad K, Ullah H, Obaidat MS, Ullah A, Munir A, Sajjad M, et al. AI-Driven Salient Soccer Events Recognition Framework for Next-Generation IoT-Enabled Environments. Institute of Electrical and Electronics Engineers (IEEE); 2021. p. 2202. (IEEE Internet of Things Journal; vol. 10).   The detailed revision is written from line 18 to line 20. |

**Reviewer 2**

| Comments | Responses |
| --- | --- |
| 1. *In the following sentence: ‘In this study, the application of these technologies within the domain of association soccer is examined, with a particular focus on predicting team strategies via team trend analysis.’ Would methodologies be a more appropriate term to use instead of technologies? The following sentence does not read correctly and does not make sense: ‘The prediction of strategies from soccer match datasets challenges.’ Please rewrite this sentence.* | Thank you for your comment. I agree that the term "methodologies" is more accurate in a broader sense. The term "methodologies" more precisely reflects the methods and processes utilized in our research, including the Elo rating system, rolling regression, and predictive modeling techniques. These changes aim to improve the accuracy and clarity of the manuscript. For better clarity, I have revised the following sentences:  Original Sentence: ‘In this study, the application of these technologies within the domain of association soccer is examined, with a particular focus on predicting team strategies via team trend analysis’.  Revised Sentence: ‘In this study, the application of these methodologies within the domain of association soccer is examined, with a particular focus on predicting team strategies via team trend analysis.’  Original Sentence: ‘The prediction of strategies from soccer match datasets challenges.’  Revised Sentence: ‘Predicting strategies from soccer match datasets is a challenging.’ |
| 2. *Introduction Line 31 – It should read ‘overlook’ not ‘overlooking’ – please change this.* | Thank you for your comment. I appreciate the opportunity to improve my paper.  Original Sentence: ‘Existing studies often focused on team formation, position, and game records for analysis, overlooking team trends.’  Revised Sentence: ‘Existing studies often focused on team formation, position, and game records for analysis, overlook team trends.’  The detailed revision is written from line 30 to line 31. |
| 3. *Methodology - There appears to be no title for the methodology section. Please add this. There is no mention of the statistical program/coding method that was used to calculate all of the models outlined within the study. Please include this.* | Thank you for your comment. You mentioned that "there appears to be no title for the methodology section." Our paper includes detailed methodologies spread across several sections, with each section focusing on a specific aspect of the analysis. The titles corresponding to the 'methodology' section are as follows: Soccer Team Trend Analysis for League Matches, Team Rank for Elo Rating, Rolling Regression for Elo Rating Trend, Identifying Upswings and Downswings, Predictive Modeling of Team Formation Strategies, Team Strategic Approach for Formation Style, and Match-Result-Based Strategy Feature Extraction. This structure was chosen to provide clear and focused discussions on each methodological component.  We also noted the absence of a mention regarding the statistical program/coding method used for all model computations in our paper. To address this, we have included the relevant information in the Experimental Settings section of the Results. The added text is as follows:  ‘All analyses and model computations were performed using Python. The parameters set during training for each proximity are as follows: batch size = 128 and epochs = 10. We used the Adam optimizer with a dropout rate of P drop = 0.1 and a learning rate of 0.01. Dropout was applied to the output of each sub-layer, and L2 regularization (weight decay) of 0.001 was applied in the fully connected layers.’  This revision aims to enhance the explanation of the statistical program and coding methods used to compute the models in our research.  The detailed revision is written from line 302 to line 306. |
| 4. *Line 115 and 119/120 repeat the same information about using the Elo rating system for teams not individuals. This only needs to be included once in this section so one of these sentences should be edited/removed. Line 141 – the manuscript has already mentioned that Elo was used in chess, so it is not required to be mentioned again here.* | Thank you for your comment. I apologize for the redundancy in the text. To address this, I have revised the paper to remove the repetitive information related to the use of the Elo rating system for teams. Specifically, I have removed the redundant information from lines 119/120, while keeping the essential discussion in line 115. Additionally, I have eliminated the repetitive mention of the Elo system's origin in chess from line 141.  This revision aims to correct the redundancy found in lines 115, 119, 120, and 141. |
| 5. *Line 188 – The authors have highlighted that the Elo-based approach addresses limitations that other ranking systems have such as the ability to consider opponent strength and the impact of home and away matches. I understand that opponent strength is considered within the RA and RB, but I am unsure how the Elo system considers the impact of home and away matches. I may have missed this point, but please can you clarify this point in the text.* | Thank you for your comment. You are correct that the Elo rating calculation considers the strength of the opposing team but does not currently account for the effects of home and away matches. I apologize for the confusion caused by the erroneous inclusion of this aspect in the explanation.  To address this issue, we have revised the manuscript to omit the incorrect reference to home and away match considerations. Below is the revised content for clarity:  Original Sentence: 'Unlike traditional rankings, the Elo-based approach addresses limitations such as the lack of opponent strength consideration and the impact of home and away matches.'  Revised Sentence: 'Unlike traditional rankings, the Elo-based approach addresses limitations by considering the strength of opponents.'  The detailed revision is written from line 183 to line 184. |
| 6. *Results Line 294 – The authors should provide reasons as to why domestic and continental club tournaments do not provide a consistent and reliable set of data, but domestic league games do.* | Thank you for your comment. I apologize for any confusion caused by the use of the term "reliable." Below are the corrected details regarding this issue:  Original Sentence: "We focused on league matches because they provide a consistent and reliable set of data, and we chose not to include matches from continental club tournaments or domestic cups."  Revised Sentence: "We focused on league matches because they provide consistent data, and decided not to include matches from continental club tournaments or domestic cup competitions."  We focused on domestic league matches for several reasons, as they provide a consistent dataset. In contrast, matches from continental club tournaments or domestic cups were excluded for the following reasons:  Domestic league matches are held regularly throughout the season, providing a structured schedule and consistent data points for analysis. Each team plays a similar number of matches under uniform conditions, allowing for balanced comparisons of performance metrics. Additionally, domestic league matches involve teams from the same league, ensuring a consistent level of competition.  In contrast, continental club tournaments and domestic cups often involve matches between teams from various divisions and leagues, leading to varied levels of competition and performance standards. Our study specifically targets the top five European leagues (England, Spain, Germany, Italy, and France). Including matches from continental tournaments or domestic cups could introduce data from leagues outside our primary focus, complicating the analysis and potentially affecting the validity of our experimental results due to a smaller dataset.  The detailed revision is written from line 289 to line 291. |
| 7. *Line 318 – the description of the requirements for each cluster is very vague. The authors should expand on these explanations to provide further insights into the tactics and strategies incorporated into each cluster: ‘For instance, Cluster 2 consisted of teams that adopted widely used strategies, whereas Cluster 1 was characterized by teams that employed less common tactics.’*   *Line 321 – The description of ‘Cluster 0’ should be moved earlier with the descriptions of cluster 1 and cluster 2.* | Thank you for your comment. As you pointed out, the explanation should be expanded to provide additional insights into the tactics and strategies integrated into each cluster. I agree that the current explanation in the paper could cause confusion. To address this, we have expanded the explanation based on our research results to include more detailed descriptions. Below, we provide a detailed explanation of the clustering process and the characteristics of each cluster based on our findings. Here are the revised:  ‘We examined the tactics primarily used in each cluster. Cluster 0 frequently employed formations such as '4-0-4-0-2' and '5-0-3-0-2'. Cluster 1 predominantly used the '4-0-2-3-1' formation. Teams in Cluster 2 mainly utilized formations like '4-0-3-0-3' and '3-0-4-0-3'. Each cluster also exhibited significant characteristics beyond tactics. For instance, Cluster 0 mainly relied on defensive strategies, evidenced by the highest defense score of 8 points according to our calculation method. Conversely, Cluster 2, with a defense score of 7 points, played more offensively compared to Cluster 0. The difference between Cluster 1 and Cluster 2 arises more from the tactical variations rather than the overall play style.’  The detailed revision is written from line 320 to line 327. |
| 8. *Table 4 and Table 5 – the different models within these tables have not been described within the methodology section of the paper. A description of any statistical models used within the study should be clearly written for the reader to understand and be able to replicate.* | Thank you for your comment. As you pointed out, the methodology section lacked explanations regarding the statistical models used. We have now included detailed descriptions of the statistical models used in our study in the 'Formation Style Prediction' section. Below are the revisions we made to our research:  ‘To analyze the impact of team formation styles on match outcomes, we employed several predictive models: MLP, Random Forest, Gradient Boosting, Logistic Regression, and Decision Tree. Each model was selected for its unique characteristics and strengths. MLP is effective at capturing nonlinear relationships in complex datasets. Random Forest enhances robustness by combining multiple decision trees and managing datasets effectively. Gradient Boosting builds trees sequentially, correcting errors from previous iterations, thereby improving accuracy in complex scenarios. Logistic Regression offers a straightforward approach for binary classification, providing insights into probabilistic outcomes and feature influences. The Decision Tree model is intuitive and interpretable, making it useful for understanding decision processes and modeling feature interactions. For comprehensive performance evaluation, these models were assessed using cross-validation and metrics such as accuracy, precision, recall, and F1 score.’  The detailed revision is written from line 365 to line 376. |
| 9. *Line 372 – ‘The superior recall of random forest in the ranking-base approach suggests its strength in accurately classifying true-positive instances, indicating its ability to effectively capture nuanced differences in the impact of strategic factors on match outcomes.’ This statement on the effectiveness of the random forest model for rank-based approaches seems to be too strong. This model had a recall of 0.72 which, while it was the best predictor compared to the other models, it still means that it is incorrect 28% of the time. This is not highly accurate, so this sentence needs to be rewritten to reflect that.* | Thank you for your comment. I agree that the original statement was too strong given the recall rate of 0.72. Although the Random Forest model performed better compared to other models, it is important to present a balanced perspective on its accuracy. Below are the revisions made to more accurately reflect the model's performance:  ‘In the ranking-based approach, the random forest model achieved a recall rate of 0.72, the highest among the models tested. However, this also indicates a 28% inaccuracy. While this demonstrates the random forest model's relative strength in classification compared to other models, it also highlights the need for further improvements to enhance accuracy. This suggests that there is still room for improvement in this model.’  The detailed revision is written from line 392 to line 396. |
| 10. *Conclusion , Figure 8a and 8b should not appear in the conclusion and need to be moved into the model evaluation section – no more new data should be presented within the conclusion.* | Thank you for your comment. As you noted, Figures 8a and 8b are currently placed in the Conclusion section, which could indeed cause confusion. Following your suggestion, I will revise the manuscript to relocate these figures to the 'Model Evaluation' section. |
