# Supplementary material for: Data-driven understanding on soccer team tactics and ranking trends: Elo rating-based trends on European soccer leagues
Source: PLoS One. 2025 Feb 5;20(2):e0318485. doi: 10.1371/journal.pone.0318485 (PMC11798493; doi:10.1371/journal.pone.0318485)
Supplement: Attachment — Submitted filename: Revision-Letter.docx [file pone.0318485.s003.docx]

**Journal Revision Letter**

Dear **reviewers**

Thank you very much for your comments and suggestions on our paper titled **“Data-driven understanding on soccer team tactics and ranking trends: Elo rating-based trends on European soccer leagues”**. The comments are very insightful and helpful in improving the quality of our paper and provide important guidance to our research. We have carefully considered and paid special attention to every comment and have responded to each one of them as detailed below. We sincerely hope that the revisions will be satisfactory for your approval.

Here is a point-by-point response to the reviewers’ comments and concerns.

**Reviewer 3**

| Comments | Responses |
| --- | --- |
| 1. *Question 1: Line 13-14，“Despite market expansion, soccer coaches’ training and tactical decisions are often influenced more by personal judgment and experience than by data-driven insights.”This description is too absolute. Is there any literature to support it?* | Thank you for your comment. "Despite market expansion, soccer coaches' training and tactical decisions are often influenced more by personal judgment and experience than by data-driven insights." I agree with your perspective and have added references to support this argument in the paper. Below is the added paper:  Rein R, Memmert D. Big data and tactical analysis in elite soccer: future challenges and opportunities for sports science. 2016 Aug 24;5(1).  The detailed revision is written from line 13 and line 14. |
| 2. *Question 2: The introduction of experimental data sources should be placed in the first half of the paper* | Thank you for your comment. I agree with your perspective and have placed the introduction of the experimental data sources in the earlier sections of the paper. Considering the importance of introducing the data sources, I have provided an explanation of their relevance to the research questions. The data is introduced immediately after presenting the research questions. Below is the added content:  This study utilized data from the top five European soccer leagues, comprising 3,652 matches, to analyze using detailed metrics such as team formations, match outcomes, and rankings.  The detailed revision is written from line 45 and line 49. |
| 3. *Question 3: It is suggested to add the introduction of football game Tactics* | Thank you for your comment. I appreciate the suggestion to include an introduction to soccer match tactics. To address this, I have added an explanation in the "Team strategic approach for formation style" section, providing an overview of soccer tactics. This includes common formations, strategies, and their impact on match outcomes. Below is the added content:  In soccer, match tactics refer to the strategic formations and play styles employed by a team to achieve specific objectives during a game. Among these tactics, the composition of the defensive line significantly influences a team's strategic direction and game management.  The 3-back formation consists of three central defenders and two wing-backs, designed to strengthen central defense while allowing wing-backs to support both offensive and defensive plays. This formation effectively utilizes wide spaces on the flanks and facilitates quick counterattacks. Common formations within this system include 3-0-5-0-2 and 3-0-4-0-3.  The 4-back formation is the most stable system, comprising four defenders to maintain a balance between defense and attack. Full-backs in this system contribute to both defensive coverage and offensive support, offering high tactical flexibility that adapts to various game scenarios. Representative formations include 4-0-4-0-2 and 4-0-3-0-3.  The 5-back formation prioritizes defensive solidity by deploying five defenders to fortify both central and wide defensive zones. While highly effective in maintaining a robust defense, this system may limit offensive build-up. Typical formations include 5-0-3-0-2 and 5-0-4-0-1.  The detailed revision is written from line 232 and line 245. |

**Reviewer 4**

| Comments | Responses |
| --- | --- |
| 1. *The introduction provides a clear overview of the significance of soccer analytics and highlights the role of big data and machine learning in advancing sports analytics. However, A brief explanation of the Elo rating system and its relevance to team performance could strengthen the context.* | Thank you for your comment. I appreciate the valuable suggestion to include a brief explanation of the Elo rating system and its relevance to team performance. In the revised manuscript, we have incorporated a concise description of the Elo rating system into the introduction section to strengthen the study's context. This explanation highlights the system's origins, its application to soccer analysis, and its utility in evaluating team performance trends. Below is the added content:  The Elo rating system, originally developed for ranking chess players, has been adapted in soccer to evaluate team performance dynamically. By updating team ratings after each match based on expected and actual outcomes, the system provides a nuanced understanding of a team's relative strength and performance trends over time. This approach offers a more comprehensive perspective compared to traditional ranking systems, making it highly relevant for analyzing team strategies and predicting future performance.  The detailed revision is written from line 27 and line 32. |
| 2. *The qualitative methodology is suitable for the study, the dual approach of count-based and rank-based methods provides a comprehensive framework for analyzing strategies but more clarity on dataset specifics (e.g., sample size, range of seasons analyzed, preprocessing steps) would enhance replicability.* | Thank you for your comment. I appreciate your valuable feedback on improving the clarity of the dataset details in the study. In the revised manuscript, we have expanded the experimental settings section to include more detailed information about the dataset. Below is the added content:  Original Sentence: “Our goal was to gather and analyze key information about team tactics from the top five European soccer leagues, namely, England, Spain, France, Italy, and Germany. We collected data from various websites, including sports-reference(https://www.sports-reference.com/), covering 3652 games played by 98 teams throughout the season. The data that we focused on included aspects such as matchday, team name, opposing team, team formation, opponent formation, match result, home/away status, GF, GA, and team rank. We focused on league matches because they provide consistent data, and decided not to include matches from continental club tournaments or domestic cup competitions.”  Revised Sentence: “Our goal was to gather and analyze key information about team tactics from the top five European soccer leagues, namely, England, Spain, France, Italy, and Germany. We collected data from various websites, including sports-reference(https://www.sports-reference.com/), covering 3652 games played by 98 teams during a single season. The data that we focused on included aspects such as matchday, team name, opposing team, team formation, opponent formation, match result, home/away status, GF, GA, and team rank. Preprocessing involved normalizing numerical variables such as goals for and against, encoding categorical features such as formations and home/away status, and addressing missing data by interpolating results based on recent team performance. We focused on league matches because they provide consistent data, and decided not to include matches from continental club tournaments or domestic cup competitions.”  The detailed revision is written from line 308 and line 318. |
| 3. *Results provides quantitative findings, such as the accuracy of predictions (85% for defensive strategies and 89% for aggressive strategies), but Lack of comparative benchmarks with other models or methods makes it difficult to evaluate the study’s innovation.* | Thank you for your comment. I appreciate your insightful suggestion regarding the need for comparative benchmarks with other models or methods to better reflect the study's outcomes. I acknowledge that incorporating such comparisons would strengthen the analysis and provide a more comprehensive understanding of the model's performance.  In this study, we focused on developing and evaluating a dual approach (count-based and ranking-based) for soccer strategy prediction. While the achieved prediction accuracies—85% for defensive strategies and 89% for offensive strategies—are respectable, the lack of publicly available datasets aligned with the study's characteristics and objectives limited the inclusion of direct comparisons with existing models or methods. We recognize the importance of comparative benchmarks to evaluate the relative performance of our approach. Future research will focus on testing our methods on standardized datasets and benchmarking against existing approaches to better assess the relative performance and innovations of the model. |
| 4. *The discussion highlights the practical implications of predictive models for coaching and game preparation, but it could delve deeper into the limitations of the study, such as potential overfitting or generalizability issues.* | Thank you for your comment. I appreciate your insightful feedback on the study's limitations, particularly the need to delve deeper into issues such as potential overfitting and generalization challenges. Thank you for the opportunity to provide further clarification, and the manuscript has been revised to include a detailed discussion of these limitations. Below is the added content:  Original Sentence: Despite its many advantages, this study has some limitations. For example, the dataset used in this study was limited to the top-five European leagues, which restricts the scope from encompassing the full spectrum of tactical variations, team compositions, and match outcomes found in a wider array of soccer leagues. Future studies should collect data from a wider variety of leagues and teams to address these limitations and undertake more comprehensive analyses. Although we predicted team strategies with relative accuracy, unexpected aspects of team formations or match outcomes were observed. We recognized trends across various games; however, not all trends were utilized in this study. Future work should incorporate all unused trends and seek to design methods for predicting team formations and match outcomes, considering the opposition level. These efforts will aim to predict a broader array of soccer strategies and develop applications for a larger number of teams, thereby enhancing the global understanding and application of strategic formations in soccer. Below is the revised content:  Revised Sentence: Despite its many advantages, this study has some limitations. The proposed model demonstrated high predictive accuracy but also exhibited several limitations. First, despite measures such as cross-validation and dropout regularization, the relatively small dataset and the complexity of the model raise the possibility of overfitting. Second, the generalizability of this study is constrained as the dataset focuses on a single season of matches from the top five European leagues. This limitation may prevent the full capture of strategic variations across seasons, leagues, or international competitions. Addressing these limitations will be a key focus of future research, which aims to expand the dataset to include multiple seasons and additional leagues, as well as to further evaluate the robustness of the model by employing independent test sets. Future studies should collect data from a wider variety of leagues and teams to address these limitations and undertake more comprehensive analyses. Although we predicted team strategies with relative accuracy, unexpected aspects of team formations or match outcomes were observed. We recognized trends across various games; however, not all trends were utilized in this study. Future work should incorporate all unused trends and seek to design methods for predicting team formations and match outcomes, considering the opposition level. These efforts will aim to predict a broader array of soccer strategies and develop applications for a larger number of teams, thereby enhancing the global understanding and application of strategic formations in soccer.  The detailed revision is written from line 500 and line 509. |
| 5. *References are diverse and relevant, covering foundational concepts and recent advancements in sports analytics, but some references appear dated; more recent studies could be incorporated to reflect current trends in machine learning and sports analytics.* | Thank you for your comment. I appreciate your suggestion to include recent studies to better reflect current trends in machine learning and sports analytics. I recognize the importance of integrating recent papers to enhance the relevance and comprehensiveness of the reference list. Below is the added content, including citations of the latest studies:    “The advent of big data and artificial intelligence (AI) has profoundly transformed sports analytics.”  Reference List:   1. Markopoulou C, Papageorgiou G, Tjortjis C. Diverse Machine Learning for Forecasting Goal-Scoring Likelihood in Elite Football Leagues. 2024 Jul 28;6(3):1762. 2. Papageorgiou G, Sarlis V, Tjortjis C. Evaluating the effectiveness of machine learning models for performance forecasting in basketball: a comparative study. 2024 Mar 24;66(7):4333.   Added Sentence:  “Recent studies have also explored the integration of machine learning in sports analytics, including its applications in soccer strategy optimization.”  Reference List:   1. Pu Z, Pan Y, Wang S, Liu B, Chen M, Ma H, et al. Orientation and Decision-Making for Soccer Based on Sports Analytics and AI: A Systematic Review. 2024 Jan 1;11(1):37. 2. Rodu J, Dejong Lempke AF, Kupperman N, Hertel J. On Leveraging Machine Learning in Sport Science in the Hypothetico-deductive Framework. 2024 Nov 14;10(1).   The detailed revision is written from line 19.  The detailed revision is written from line 66 and line 68. |
| 6. *The study’s focus on trend-based strategy prediction is novel and valuable, but the lack of practical implementation diminishes its immediate impact.* | Thank you for your comment. I appreciate your feedback regarding the lack of practical implementation and its impact on the immediate applicability of the study. Thank you for recognizing the value of the trend-based strategy prediction approach, and I agree that practical application would enhance the contribution of this research.  While this study focuses on establishing a theoretical framework for trend-based strategy prediction, future research will prioritize practical implementation. This will include developing real-time tools for coaches and analysts, testing in real-world scenarios, and customizing the approach for various competitive contexts. These steps aim to bridge the gap between theoretical insights and actionable applications, thereby enhancing the immediate impact of the proposed methodology. |
